# Increased Risk for Dementia in Patients With Inflammatory Bowel Disease: A Systematic Review and Meta-Analysis of Population-Based Studies

**DOI:** 10.3389/fneur.2022.813266

**Published:** 2022-05-13

**Authors:** Mengsi Liu, Dongxiu Li, Xia Hong, Zhen Sun

**Affiliations:** ^1^Hengyang Medical School, University of South China, Hengyang, China; ^2^Department of Nursing, Fujian Health College, Fuzhou, China

**Keywords:** dementia, neurodegenerative disease, inflammatory bowel disease, risk factor, comorbidity

## Abstract

**Background:**

Mounting evidence suggests that there may be a causal relationship or common pathogenic pathway between inflammatory bowel disease (IBD) and dementia. However, inconsistent results have emerged from epidemiological studies. We therefore conducted this review to clarify the relationship between IBD and dementia.

**Methods:**

We systematically searched PubMed, Web of Science, Embase, and Cochrane library to identify all studies exploring the relationship between IBD and dementia published as of September 2021. Risk estimates were pooled using both fixed and random-effects models.

**Results:**

Six studies involving 2,334,472 subjects were included. Pooled results suggested that the risk of developing dementia significantly increased after IBD diagnosis (HR = 1.27, 95% CI: 1.10–1.47, *P* = 0.001), which did not vary by age, gender, dementia subtype, or IBD subtype. Whereas, the dementia incidence before IBD diagnosis and the comorbidity rate of dementia in IBD patients were similar to those without IBD (HR = 0.92, 95% CI: 0.68–1.25; 0.82, 95% CI: 0.64–1.06, respectively). However, current evidence was insufficient to establish a causal relationship.

**Conclusion:**

This study shows an unidirectional association between IBD and dementia; patients with IBD have an increased risk of dementia, and it may be beneficial to develop individualized dementia screening strategies for this population. Future research needs to further investigate whether effective therapies of IBD can reduce this risk and pathophysiological mechanisms of the association.

## Introduction

Inflammatory bowel disease (IBD), consisting mainly of Crohn's disease (CD) and ulcerative colitis (UC), is a chronic inflammatory disease of the intestine, the exact etiology of which remains unknown (1). Current evidence suggests that IBD pathogenesis may be related to the host genetic susceptibility, environmental factors, dysbiosis of gut microbiota, and abnormal immune system (2). IBD primarily affects the intestinal tract causing abdominal pain, diarrhea, and blood in stool, but it is not confined to the gut and extraintestinal manifestations can often be observed (3, 4). With its global prevalence continuing to increase, IBD places a huge economic burden on health systems and has become a worldwide health care challenge (5).

Dementia is a syndrome characterized by chronic or progressive cognitive decline, caused by the interaction of genes, lifestyle, and environmental factors, and is usually irreversible (6). Dementia includes several subtypes, with Alzheimer's disease (AD) being the most commonly diagnosed and the most prevalent neurodegenerative disease (7). Similar to IBD, the incidence of dementia is continuously increasing and imposes a heavy social and economic burden (8, 9). Already more than 55 million people worldwide are living with dementia and the number is expected to increase at an alarming rate of 10 million per year (10). To make matters worse, there is no effective treatment that can cure dementia (11).

There is growing evidence of the bidirectional link between gastrointestinal tract and central nervous system, also known as the “gut-brain axis” theory (12). Recent studies have found that alterations in gut microbiota and physiology have an impact on host cognitive behavior, and that disturbances in gut flora may increase the incidence of neurodegenerative diseases (13). Furthermore, neuroinflammation plays an important role in different types of dementia (6, 14). A prospective study has shown an association between systemic inflammation in mid-life and brain volume loss in late-life, suggesting that elevated inflammatory markers *in vivo* may cause neuroinflammation and thus have a pathogenic effect on cognitive aging and neurodegeneration (15). These studies indicate that there may be a common pathogenic pathway or causal relationship between IBD and dementia.

Identifying people at high risk of dementia and therefore developing risk management strategies and early diagnosis and intervention are critical to reducing the risk and slowing the progression of dementia; hence, the association between dementia and IBD has attracted growing interest from the scientific community and health care physician. Most studies have confirmed the correlation between IBD and subsequent risk of dementia, but this correlation was not observed in certain populations. Because of the conflicting results, we conducted this meta-analysis and systematic review to clarify the relationship between IBD and dementia, taking into account the effects of study design, age, gender, and disease subtype.

## Materials and Methods

The present systematic review and meta-analysis was performed according to Preferred Reporting Items for Systematic Reviews and Meta-Analyses (PRISMA 2020) guidelines (16). The protocol for this study is not registered.

### Search Strategy

Two reviewers extensively searched the PubMed, Embase, Web of Science, and Cochrane library for relevant papers published from inception date to September 2021. Search strategies were developed based on Medical Subject Headings terms (such as dementia, inflammatory bowel disease, Crohn disease, “colitis, ulcerative”) in combination with free terms (such as dementias, demention, amentia, bowel diseases inflammatory, Crohn's enteritis, idiopathic proctocolitis), without language restrictions or use of filters. We also manually searched the reference lists of included studies and related reviews to identify potentially eligible studies. Detailed search strategies for each database are provided in the Supplementary Table A1.

### Eligibility Criteria

Reports that met all of the following criteria would be considered for inclusion: (a) IBD and dementia were identified according to standard diagnostic criteria or according to disease classification or prescription codes in the patient medical record database; (b) study results reported hazard ratio (HR), risk ratio, or odds ratio with their corresponding 95% confidence intervals (CIs) for the association between IBD (CD and/or UC) and any dementia subtype, or provided sufficient data to calculate these ratios; and (c) the study design was case-control, cohort, or cross-sectional;

Reviews, meta-analyses, animal studies, commentaries, case reports or series with sample sizes of <10, conference abstracts published in full elsewhere, and single arm studies would be excluded. We would examine the period and location of population selection for each report, and for reports containing overlapping data, we included reports with larger sample sizes.

### Study Selection and Data Extract

Two authors independently reviewed the titles and abstracts of initially retrieved records according to inclusion and exclusion criteria. Potentially eligible reports were assessed by full-text reading. Reasons for exclusion after full-text reading would be recorded. Any disagreements were resolved by consensus through discussion.

For the final included studies, data were extracted including: first author, year of publication, region, age, gender, study period, study design, source of subjects, sample size, identification methods for IBD and dementia, confounders being matched or adjusted for, and duration of follow-up. Data was recorded by one author and checked by another.

### Study Quality Assessment

For cohort and case-control designs, the quality of included studies was assessed using the Newcastle-Ottawa Scale (NOS), which includes eight specific quality items in three domains: selection, comparability, and exposure/outcome (17). All items were given one star except for comparability, which could be assigned a maximum of two stars. The score ≥7 was considered high quality, otherwise low quality. The median/mean follow-up period ≥5 years or maximum follow-up period ≥10 years was considered enough. For the cross-sectional study, we used the Agency for Healthcare Research and Quality (AHRQ) instrument to assess quality, with a total of eleven items (18). One point was given for a 'yes' answer to an item, and no points for “no” or “unclear”. Studies scoring 0–8 and 9–11 were considered low and high-quality studies, respectively.

### Statistical Analysis

In this study, all data analyses were performed using Stata MP/16.0. The inverse variance method of DerSimonian and Laird was used to pool the risk estimates and corresponding 95% CIs (19). For studies where both unadjusted and adjusted risk estimates were provided, we would extract the adjusted risk estimates for analysis. The HR, risk ratio, and odds ratio were considered equivalent because of the relatively low incidence of dementia. Cochran's *Q* test and Higgin's *I*^2^ statistics were used to examine the heterogeneity of the included studies (20). Heterogeneity was considered significant if *I*^2^ > 50% or *P* < 0.1, and then we would report the result of random-effects model and look for sources of heterogeneity by examining study design and characteristics, otherwise the result of fixed-effects model was reported. A *P*-value < 0.05 was considered to be statistically significant. The sensitivity of pooled results was assessed by excluding one study at a time and then repeating the analysis. In addition, we also assessed the robustness by comparing the results of the random-effects and fixed-effects models. Subgroup analyses were performed according to the temporal relationship between dementia and IBD diagnosis (dementia before IBD, dementia after IBD, comorbidity of dementia in IBD), dementia subtype (AD, other dementia types), IBD subtype (UC, CD), sex, age (<65, >65 years). The Begg's and Egger's tests were used to assess publication bias. When the *P*-values for both ≥0.05, there was no publication bias, otherwise the impact of potentially unpublished studies was assessed by using the trim-and-fill method.

## Results

**Figure 1:**
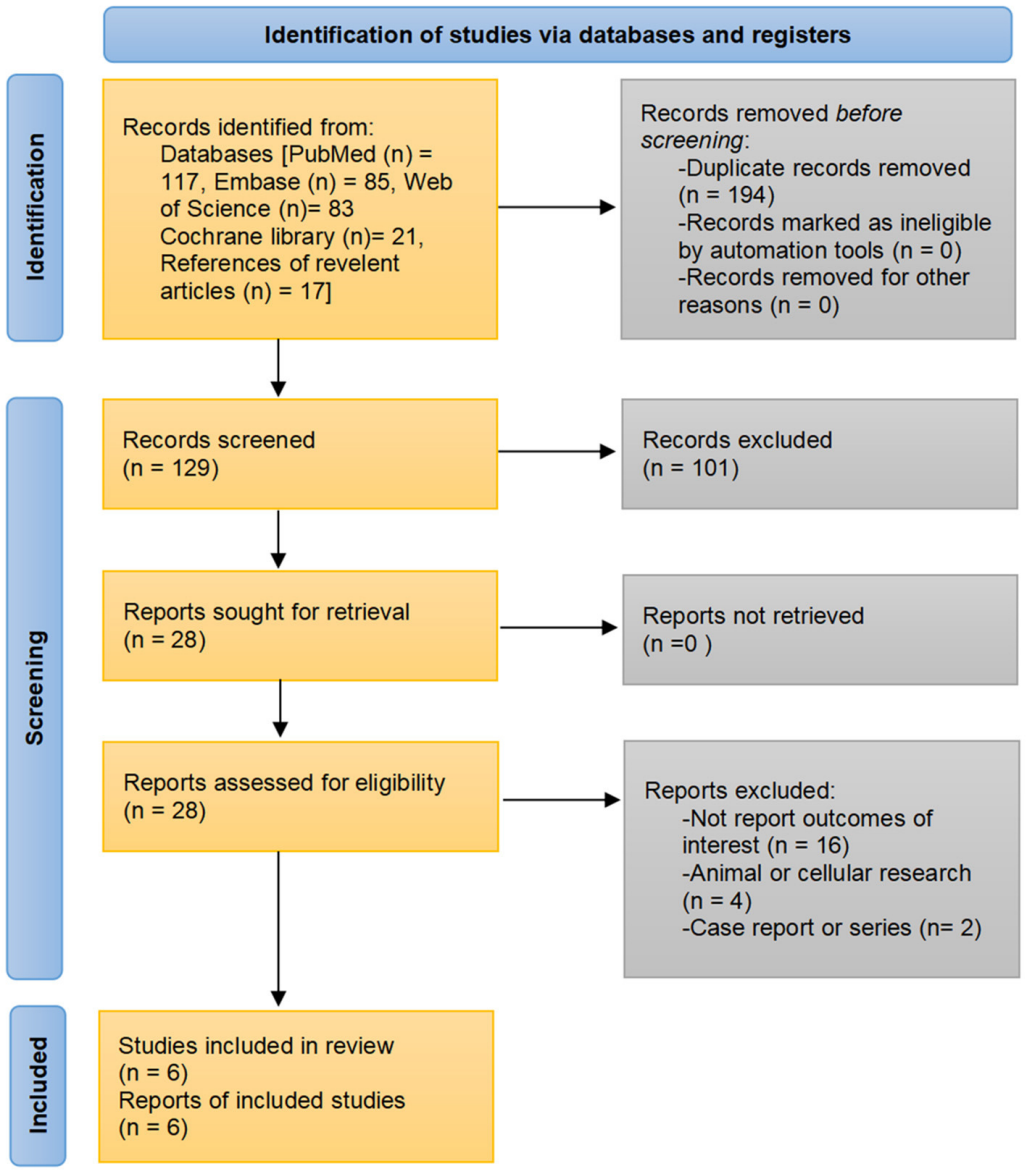
Flowchart of study selection.

A total of 323 records were identified through the initial search strategies. After excluding duplicates and reviewing titles, abstracts, and/or full texts, six population-based studies fulfilled eligibility criteria to be included in the analysis (21–26). The detailed search process is shown in Figure 1.

### Study Characteristic

The six included studies were all population-based and comprised a total of 2,334,472 subjects, of which 132,445 were patients with IBD. All studies were cohort studies except one, which was cross-sectional design (24). Five studies assessed the risk of developing dementia after the IBD diagnosis (21–23, 25, 26), and two studies assessed the incidence of dementia before the IBD diagnosis and the dementia comorbidity in patients with IBD, respectively (23, 24). Diagnosis of IBD and dementia was based on diagnostic codes from medical records databases, such as the International Classification of Diseases codes and related drug prescriptions. The proportions of men and women in included studies were similar, while the median/mean age varied between studies, ranging from 36 to 71 years old. Three out of the five cohort studies had a follow-up of 15 years or more (21, 22, 25), one had 5 years (26), and one did not provide follow-up (23). All studies matched and/or adjusted for some confounding factors such as age, gender, and comorbidities. Detailed information for each study is presented in Table 1.

**Table 1:** Characteristics of studies investigating the relationship between IBD and dementia.

**References**	**Country**	**Period**	**Study design**	**Mean/median age-year**.	**Population**	**IBD**	**Non-IBD**	**Diagnosis of IBD**	**Diagnosis of dementia**	**Matching/Adjustment confounder**	**Follow-up, year**	**NOS score**
Zhang et al. (21)	Taiwan, China	1998–2011	Cohort study	IBD+: 60.6; IBD–: 60.6	Health Insurance Database	1,742	17,420	ICD	ICD	Age, sex, enrolment time, dementia-related medical comorbidities, income level, and urbanization level of residence, Charlson Comorbidity Index score, all-cause clinical visits	16	9
Zingel et al. (22)	Germany	1995–2014	Cohort study	IBD+: 70.9; IBD–: 71.0	Disease Analyzer Database	3,850	3,850	ICD	ICD	Age, sex, enrolment time, health insurance type, comorbidities	15	8
Bernstein et al. (23)	Canada	1987–2018	Cohort study	IBD+: 36; IBD–: NP	IBD Epidemiology Database	9,247	85,691	ICD	ICD	Age, sex, residential area	NP	7
Bahler et al. (24)	Switzerland	2014	Cross-sectional study	IBD+: 56; IBD–: 44	Insurance Group	4,791	1,114,638	Drug Prescription	ATC classification system	Age, sex, language area, type of insurance, urbanization, residential area	NA	10^a^
Sand et al. (25)	Denmark	1977–2018	Cohort study	NP	Nationwide population	88,985	884,108	NP	NP	Age, sex, residential area, Charlson Comorbidity Index Score, comorbidities	IBD+: median 12.3, IBD–: median 12.7	9
Kim et al. (26)	Korea	2012–2017	Cohort study	IBD+: 55.4, IBD–: 55.4	Health Insurance Database	24,830	99,320	ICD	ICD, Drug Prescription	Age, sex, residential area, Charlson Comorbidity Index	5	9

*IBD, Inflammatory bowel disease; ICD, International Classification of Diseases; ACT, Anatomical Therapeutic Chemical; NP, not applicable; NA, not reported; NOS, Newcastle–Ottawa Scale*.

a *Agency for Healthcare Research and Quality score*.

All cohort studies were high quality population-based studies with appropriate subject selection, good comparability, and adequate outcome assessment (Supplementary Table A2). The cross-sectional study by Bähler et al. was also a high-quality study with a AHRQ score of 10, as all quality items were met, except for the item on follow-up, which was not available because of the cross-sectional design (24).

### The Overall Association Between IBD and Dementia

**Figure 2:**
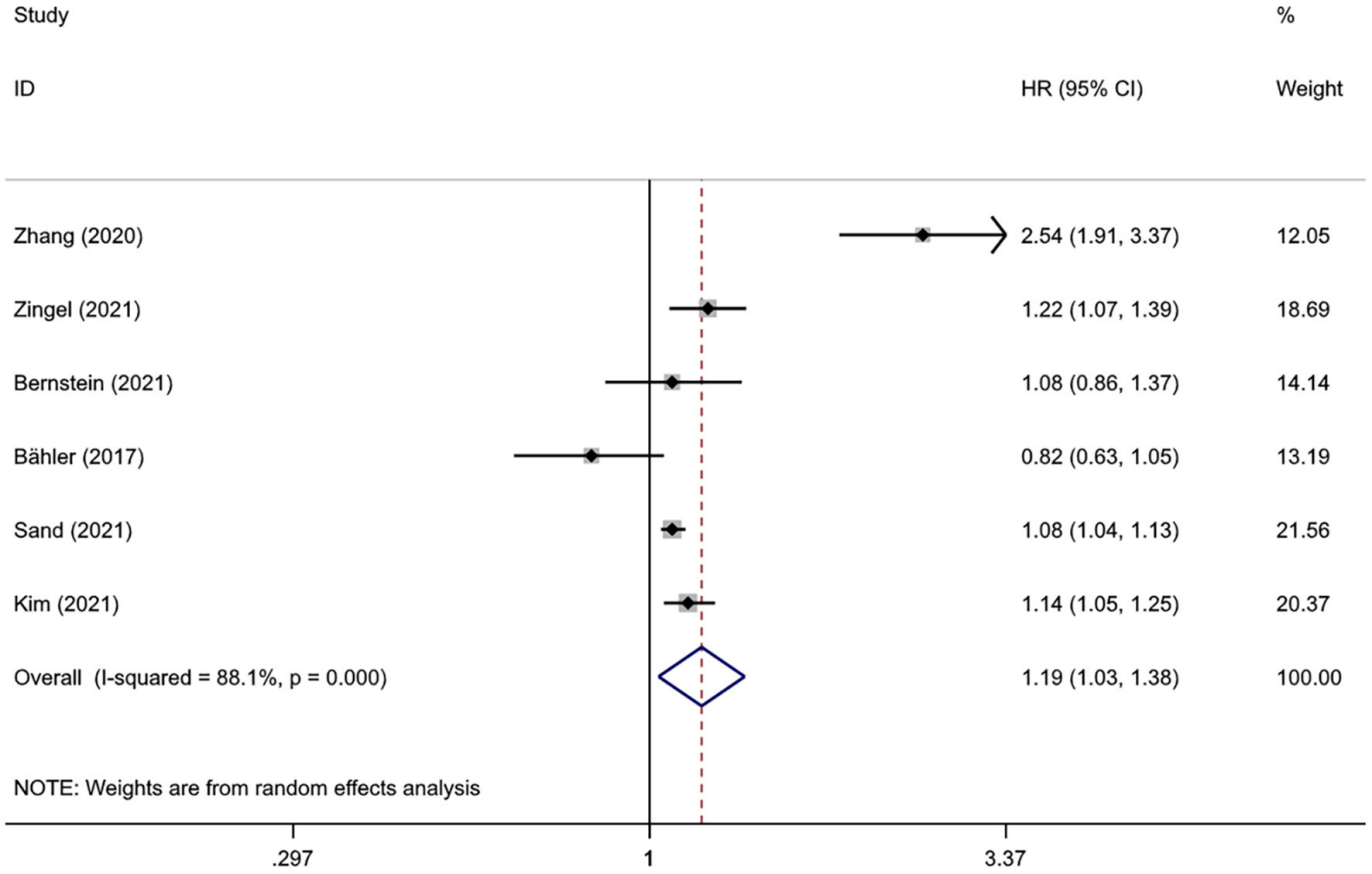
The overall association between inflammatory bowel disease and dementia.

Pooled results from six studies involving 2,334,472 subjects suggested a statistical association between IBD and dementia (HR = 1.19, 95%CI: 1.03–1.38, *P* = 0.018), with high heterogeneity (*I*^2^ = 88.1%, *P* < 0.001) (Figure 2). Heterogeneity decreased after excluding the study from Taiwan (*I*^2^ = 54.5%, *P* = 0.066), and the statistical significance of the association between IBD and dementia remained (HR = 1.10, 95%CI: 1.02–1.19, *P* = 0.014).

### Subgroup Analyses

Subgroup analysis was conducted based on the temporal relationship between dementia and IBD diagnosis, dementia subtype, IBD subtype, gender, and age. There was only one study assessing the incidence of dementia prior to the diagnosis of IBD and one assessing the dementia comorbidity in patients with IBD; given the power of pooled analysis, the subgroup analysis for dementia subtype, IBD subtype, age, and sex was limited to the group of dementia risk after IBD diagnosis.

**Figure 3:**
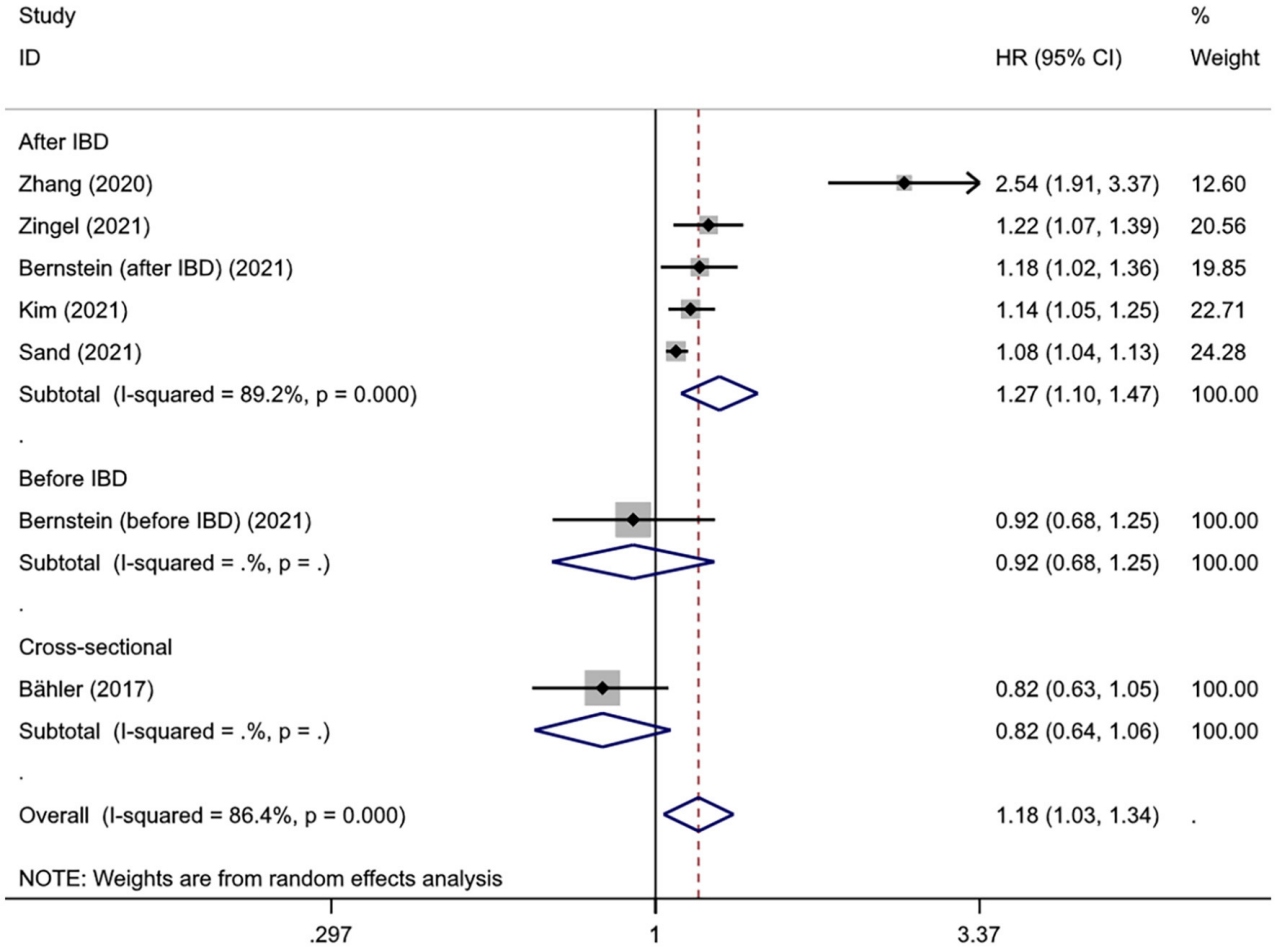
The results of temporal relationship between inflammatory bowel disease and dementia.

**Figure 4:**
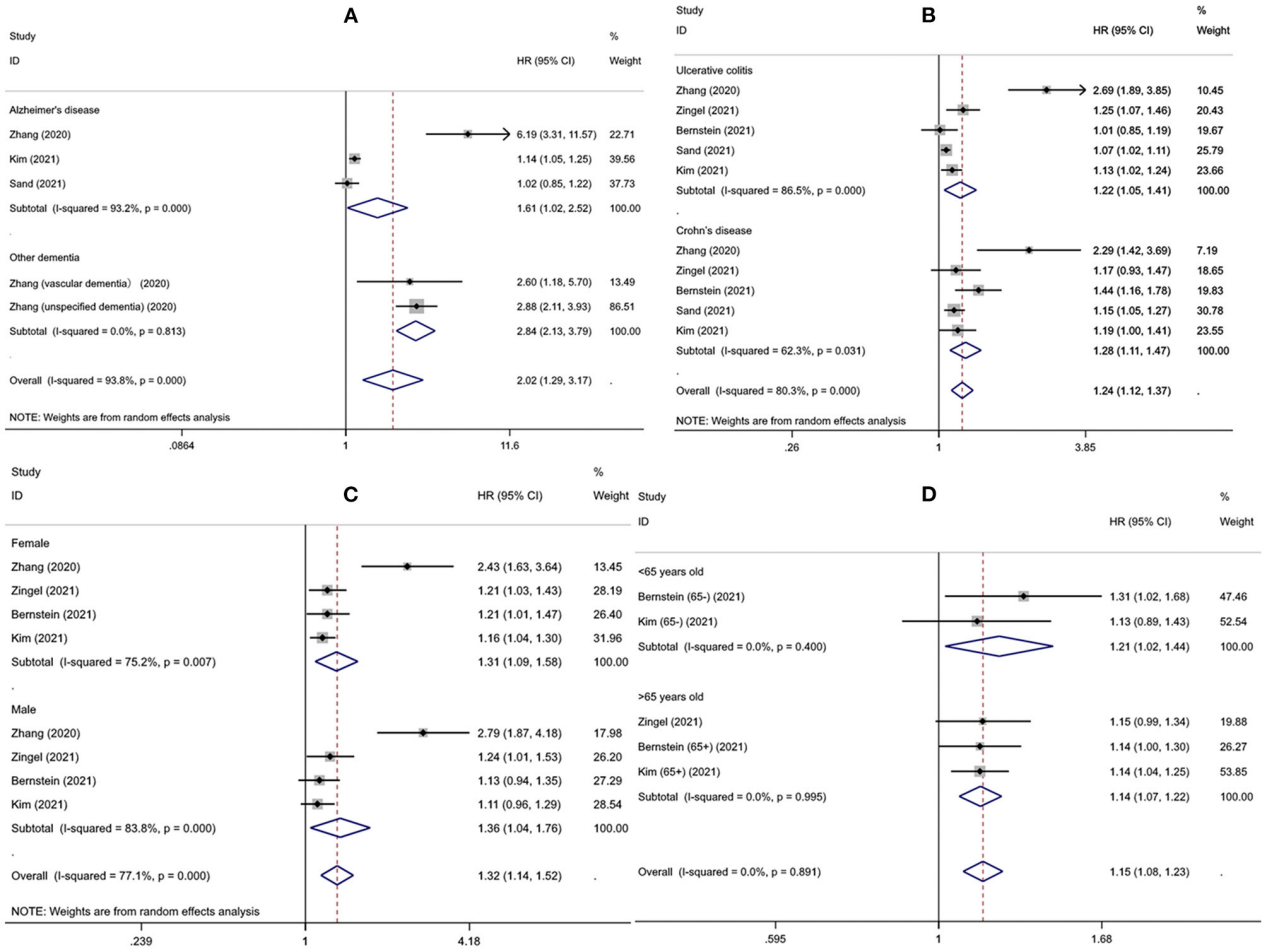
The risk of developing dementia for different subgroups **(A)** dementia subtypes **(B)** inflammatory bowel disease subtypes **(C)** gender **(D)** age.

Interestingly, subgroup analysis revealed that the risk of developing dementia significantly increased after IBD diagnosis (HR = 1.27, 95% CI: 1.10–1.47, *P* = 0.001), whereas such risk prior to IBD diagnosis and the comorbidity rate of dementia in IBD patients were slightly lower compared to controls (HR = 0.92, 95% CI: 0.68–1.25; 0.82, 95% CI: 0.64–1.06, respectively) (Figure 3). There was substantial heterogeneity in the pooled result for studies reporting IBD and subsequent risk of dementia (*I*^2^ = 89.2%, *P* < 0.001), which decreased after excluding the study from Taiwan (*I*^2^ = 35.6%, *P* = 0.198) and the significance of the result remained (HR = 1.10, 95% CI: 1.07–1.14, *P* < 0.001). There were no obvious differences in subgroups by age, sex, IBD subtype, dementia subtype; in some cases there was high heterogeneity, but all reduced to <50% after excluding the study conducted in Taiwan and remained statistically significant (Table 2, Figure 4).

**Table 2:** Results of meta-analysis of the association between IBD and dementia.

**Subgroup**	**Studies (*n*)**	**HR**	**95%CI**	***P*_overall effect_**	**Heterogeneity (*I*^2^, *P*_Q_)**
**Temporal relationship (after IBD)**	5	1.27	1.10–1.47	0.001	89.2%, <0.001
***Dementia subtype***					
Alzheimer's disease	3	1.61	1.02–2.52	0.04	93.2%, <0.001
Other dementia	1	2.84	2.13–3.79	<0.001	0.0%, 0.813
subtypes					
***IBD subtype***					
Ulcerative colitis	5	1.22	1.05–1.41	0.009	86.5%, <0.001
Crohn's disease	5	1.28	1.11–1.47	0.001	62.3%, 0.013
***Gender***					
Female	4	1.31	1.09–1.58	0.004	75.2%, 0.007
Male	4	1.36	1.04–1.76	0.022	83.8%, <0.001
***Age***					
<65 years old	2	1.21	1.02–1.44	0.028	0.0%, 0.400
≥65 years old	3	1.14	1.07–1.22	<0.001	0.0%, 0.995
**Temporal relationship (before IBD)**	1	0.92	0.68–1.25	0.591	NA
**Temporal relationship (comorbidity)**	1	0.82	0.64–1.06	0.128	NA

*IBD, Inflammatory bowel disease; HR, hazard ratio; NA, not applicable*.

### Sensitivity Analysis and Publication Bias

To assess the stability of the combined results, sensitivity analyses were conducted by excluding one study at a time and checking the influence of each study on pooled results. The results showed that the effect estimates of all groups were consistent and stable when any single study was omitted. In addition, the results from the random-effects and fixed-effects models were consistent in all pooled analyses. However, the statistical significance of the pooled results disappeared when certain studies were excluded in the AD, UC, age <65 years, and male subgroups.

The *P*-values for the Begg' and Egger' tests were 0.386 and 0.362, respectively, indicating no publication bias.

## Discussion

After pooling data from over 2.3 million participants, our results suggested that IBD is associated with an increased incidence of dementia. Patients with IBD had a 1.23-times higher risk of developing dementia, whereas dementia incidence prior to IBD diagnosis and the comorbidity rate of dementia in IBD patients were similar compared with non-IBD controls. No dementia subtype, IBD subtype, gender, or age- dependent association was observed. However, sensitivity analyses indicated that the statistical significance of association between IBD and dementia risk would disappear when certain studies were excluded in the AD, UC, age <65 years, and male subgroups, which may be attributed to the relatively small sample size and therefore lack of statistical power.

Interestingly, the high heterogeneity shown in subgroup analysis was all significantly reduced after excluding the study from Taiwan (21). The HR for the risk of dementia in patients with IBD under different conditions were close to or exceeded 2.5 in the Taiwan cohort, whereas none of the studies from other regions exceeded 1.6. After reviewing the study baselines and statistical methods of Taiwan and other regions, no sources of clinical or methodological heterogeneity were identified that might explain the statistical heterogeneity in the pooled results. One potential reason is differences in levels of health care; except for the study of Taiwan region, all other studies were from developed countries, and the well-established health care systems in developed countries may allow for more effective IBD management, thereby reducing the impact on the central nervous system. The Korean study found that the use of IBD-related medications such as immunomodulators and anti-tumor necrosis factors is associated with decreased risk of developing AD in univariable analysis, and a study from the United States came to the same conclusion (26, 27). There is already data suggesting that IBD-related biologicals are a biologically plausible approach to preserve cognitive by inhibiting neuroinflammation (28). However, the impact of regional differences in drug prescribing, healthcare policies, and clinical practice is difficult to quantify, so that effective therapies of IBD help to reduce the dementia risk is only a hypothesis and needs to be validated in future randomized controlled trials. In addition, lifestyle such as diet and exercise may also be a source of heterogeneity.

The exact mechanism for the interaction between IBD and dementia remains unknown, and current evidence suggests that IBD may increase the risk of developing dementia through several pathways. IBD is a chronic inflammatory disease involving digestive tract and other organs; despite achieving the therapeutic goal of sustained remission, histological activity, microscopic inflammation, and extraintestinal symptoms can still be found, which leads to an increased systemic inflammatory burden (29–31). Furthermore, there is a growing body of experimental and clinical data highlighting the influence of the gut-brain axis on the onset and development of neurodegenerative diseases, where the gut microbiota plays a critical role (32, 33). The dysbiosis of gut microbiome and disruption of intestinal epithelial barrier caused by IBD can facilitate the transfer of pro-inflammatory cytokines and gut microbial-derived neurotoxic metabolites through the autonomic nervous system and impaired blood-brain barrier into the central nervous system, therefore leading to neuronal damage and neuroinflammation (34–38). Neuroinflammation plays an important role in the pathology of dementia, especially the AD subtype, and recent studies have shown chronic inflammation is a driver of neurocognitive decline (39–44).

The current studies emphasized the unidirectional association between IBD and subsequent dementia onset; consistency in these studies were shown, and as discussed above, there appears to be biological plausibility that IBD causes dementia. However, current studies did not sufficiently consider temporality; dementia is a slow-progressing disease with an incubation period of several years, so reverse causality and the result of co-exposure are possible. Studies in Taiwan and Korea found that dementia risk seemed to accelerate over time with chronic course of IBD diagnosis (21, 26), but stratified analyses based on follow-up time are needed to verify the correlation at different time intervals. In addition, the strength of the association is relatively weak.

Although further clarification is needed on the causal relationship between IBD and dementia, current data suggest that IBD onset is associated with subsequent dementia diagnosis, whether as a risk factor or prodromal symptom. Moreover, the study in Taiwan indicated that the average age of dementia onset was 7 years younger in IBD patients than in controls (21). Currently, the global disease burden of IBD continues to rise with increasing average life expectancy of the world population, and rapid aging will expose more people to the threat of dementia (5, 8). Due to the efficacy of available treatments for dementia remaining unsatisfactory, it is important to identify people at high risk of dementia and patients with early dementia for timely prevention intervention (45). Current evidence suggests that care physicians should be alert to the future dementia risk among patients with IBD and provide support and education to patients and their families. Whether effective inflammation control after IBD diagnosis can provide additional benefits for preventing future dementia deserves further investigation. Moreover, a cohort study from Italy found a significantly higher risk of dying from AD in patients with UC compared to the general population, further emphasizing the importance of monitoring AD occurrence and taking risk reduction measures in patients with IBD (46).

There are some limitations in this study to note. First, there was only one study exploring the dementia incidence prior to the IBD diagnosis and one cross-sectional study exploring the dementia comorbidity among patients with IBD, so it is unclear whether there were consistent trends across regions and ethnicities. Moreover, there was only one study that assessed the relationship between IBD and dementia subtypes other than AD. Second, IBD is most commonly diagnosed in younger adults, but due to limited available data, age was divided into only two subgroups (<65 and >65 years). The characteristics of dementia risk in younger people with IBD are unclear. Third, the Begg' and Egger' tests indicate that available studies are not subject to publication bias, but the smaller number of eligible studies may limit the ability to test, so there is still a potential risk of publication bias. Several limitations of the included studies also need to be noted. Despite the large sample sizes in the included studies, the study data were all obtained from medical or insurance record databases; these data were not collected based on specific research questions, so the effects of some potential confounding factors such as physical activity, smoking, alcohol consumption, body mass index, or medications associated with dementia risk, were not adjusted/matched. In addition, some studies did not consider surveillance bias and competing risks. Moreover, there is time error in the diagnosis of IBD and dementia, such as delay in diagnosis, which is one of the reasons why causality cannot be concluded. Finally, the use of diagnostic codes to identify diseases suffers from inevitable underdiagnosis and misclassification.

In conclusion, our study demonstrates that there is a correlation between IBD and dementia, and people with IBD have an increased risk of developing dementia, regardless of gender, age, IBD subtype, and dementia subtype. Education and dementia screening for individuals with IBD to achieve early diagnosis and improve quality of life is worth considering. However, current evidence does not support drawing a conclusion about causality. Future studies from different regions and ethnicities are needed to validate this association and further elucidate the precise pathological mechanisms underlying the association between IBD and dementia; interventions on pathogenic mechanism may help develop new approaches to dementia treatment.

## Data Availability Statement

The original contributions presented in the study are included in the article/Supplementary Material, further inquiries can be directed to the corresponding author/s.

## Author Contributions

ZS: conception and design, development of methodology, critical review of the manuscript, and study supervision. ML and DL: database search, literature review, and data extraction. DL and XH: statistical analysis. ZS and ML: interpretation of data. ML, DL, and XH: drafting of manuscript. The final version of the manuscript was approved by all authors.

## Conflict of Interest

The authors declare that the research was conducted in the absence of any commercial or financial relationships that could be construed as a potential conflict of interest.

## Publisher's Note

All claims expressed in this article are solely those of the authors and do not necessarily represent those of their affiliated organizations, or those of the publisher, the editors and the reviewers. Any product that may be evaluated in this article, or claim that may be made by its manufacturer, is not guaranteed or endorsed by the publisher.
