# Supplementary material for: Increased Risk for Dementia in Patients With Inflammatory Bowel Disease: A Systematic Review and Meta-Analysis of Population-Based Studies
Source: Front Neurol. 2022 May 13;13:813266. doi: 10.3389/fneur.2022.813266 (PMC9135967; doi:10.3389/fneur.2022.813266)
Supplement: Supplementary file 1 [file Data_Sheet_1.docx]

| **Table A1.** Search strategy for each database: | |
| --- | --- |
| **PubMed** | (("Inflammatory Bowel Diseases"[Mesh] or Inflammatory Bowel Disease or Bowel Diseases, Inflammatory) OR ("Crohn Disease"[Mesh] or Crohn's Enteritis or Regional Enteritis or Crohn's Disease or Crohns Disease or Inflammatory Bowel Disease 1 or Enteritis, Granulomatous or Granulomatous Enteritis or Enteritis, Regional or Ileocolitis or Colitis, Granulomatous or Granulomatous Colitis or Ileitis, Terminal or Terminal Ileitis or Ileitis, Regional or Regional Ileitides or Regional Ileitis) OR ("Colitis, Ulcerative"[Mesh] or Idiopathic Proctocolitis or Ulcerative Colitis or Colitis Gravis or Inflammatory Bowel Disease, Ulcerative Colitis Type)) AND ("Dementia"[Mesh] or Dementias or Amentia or Amentias or Senile Paranoid Dementia or Dementias, Senile Paranoid or Paranoid Dementia, Senile or Paranoid Dementias, Senile or Senile Paranoid Dementias or Familial Dementia or Dementia, Familial or Dementias, Familial or Familial Dementias) |
| **Embase** | Sources Embase, MEDLINE  ('inflammatory bowel disease'/exp OR 'inflammatory bowel diseases':ab,ti OR 'ulcerative colitis'/exp OR 'chronic ulcerative colitis':ab,ti OR 'colitis ulcerativa':ab,ti OR 'colitis ulcerosa':ab,ti OR 'colitis ulcerosa chronica':ab,ti OR 'colitis, mucosal':ab,ti OR 'colitis, ulcerative':ab,ti OR 'colitis, ulcerous':ab,ti OR 'colon, chronic ulceration':ab,ti OR 'histiocytic ulcerative colitis':ab,ti OR 'mucosal colitis':ab,ti OR 'ulcerative colorectitis':ab,ti OR 'ulcerative procto colitis':ab,ti OR 'ulcerative proctocolitis':ab,ti OR 'ulcerous colitis':ab,ti) AND ('dementia':ab,ti OR 'amentia':ab,ti OR 'demention':ab,ti) |
| **Web of Science** | TS=(Inflammatory Bowel Diseases or Inflammatory Bowel Disease or Bowel Diseases, Inflammatory Crohn Disease or Crohn's Enteritis or Regional Enteritis or Crohn's Disease or Crohns Disease or Inflammatory Bowel Disease 1 or Enteritis, Granulomatous or Granulomatous Enteritis or Enteritis, Regional or Ileocolitis or Colitis, Granulomatous or Granulomatous Colitis or Ileitis, Terminal or Terminal Ileitis or Ileitis, Regional or Regional Ileitides or Regional Ileitis OR Colitis, Ulcerative or Idiopathic Proctocolitis or Ulcerative Colitis or Colitis Gravis or Inflammatory Bowel Disease, Ulcerative Colitis Type) AND TS=(Dementia or Dementias or Amentia or Amentias or Senile Paranoid Dementia or Dementias, Senile Paranoid or Paranoid Dementia, Senile or Paranoid Dementias, Senile or Senile Paranoid Dementias or Familial Dementia or Dementia, Familial or Dementias, Familial or Familial Dementias or demention) |
| **Cochrane library** | #1        MeSH descriptor: [Dementia] explode all trees  #2        Dementias, Familial or Familial Dementia or Familial Dementias or Dementia, Familial or Dementias or Amentia or Amentias or Senile Paranoid Dementia or Paranoid Dementias, Senile or Paranoid Dementia, Senile or Senile Paranoid Dementias or Dementias, Senile Paranoid  #3        #1 or #2  #4        MeSH descriptor: [Inflammatory Bowel Diseases] explode all trees  #5        Inflammatory Bowel Disease or Bowel Diseases, Inflammatory  #6        #4 or #5  #7        MeSH descriptor: [Crohn Disease] explode all trees  #8        Crohns Disease or Crohn's Enteritis or Regional Enteritis or Inflammatory Bowel Disease 1 or Crohn's Disease or Granulomatous Colitis or Colitis, Granulomatous or Granulomatous Enteritis or Enteritis, Regional or Enteritis, Granulomatous or Ileitis, Terminal or Ileitis, Regional or Terminal Ileitis or Regional Ileitides or Regional Ileitis or Ileocolitis  #9        #7 or #8  #10       MeSH descriptor: [Colitis, Ulcerative] explode all trees  #11       Idiopathic Proctocolitis or Ulcerative Colitis or Colitis Gravis or Inflammatory Bowel Disease, Ulcerative Colitis Type  #12       #10 or #11  #13       #6 or #9 or #12  #14       #3 and #13 |

| **Table A2.** The quality assessment of included cohort studies | | | | | | | | | |
| --- | --- | --- | --- | --- | --- | --- | --- | --- | --- |
| **Study (cohort)** | **Representativeness of exposed cohort** | **Selection of non-exposed cohort** | **Ascertainment of exposure** | **Outcome not present before study** | **Comparability** | **Assessment of outcome** | **Follow-up long enough** | **Adequacy of follow up** | **Quality score** |
| **Zhang** | ★ | ★ | ★ | ★ | ★★ | ★ | ★ | ★ | 9 |
| **Zingel** | ★ | ★ | ★ | ★ | ★☆ | ★ | ★ | ★ | 8 |
| **Bernstein** | ★ | ★ | ★ | ★ | ★☆ | ★ | ☆ | ★ | 7 |
| **Sand** | ★ | ★ | ★ | ★ | ★★ | ★ | ★ | ★ | 9 |
| **Kim** | ★ | ★ | ★ | ★ | ★★ | ★ | ★ | ★ | 9 |
